# Application of structured statistical analyses to identify a biomarker predictive of enhanced tralokinumab efficacy in phase III clinical trials for severe, uncontrolled asthma

**DOI:** 10.1186/s12890-019-0889-4

**Published:** 2019-07-17

**Authors:** Mattis Gottlow, David J. Svensson, Ilya Lipkovich, Monika Huhn, Karin Bowen, Peter Wessman, Gene Colice

**Affiliations:** 1 Biometrics and Information Sciences, AstraZeneca, Pepparedsleden 1, SE-431 83 Mölndal, Sweden; 2 IQVIA, 4820 Emperor Blvd, Durham, NC 27703 USA; 3 Present Address: Eli Lilly and Company, Lilly Corporate Center, Indianapolis, Indiana 46285 USA; 4 Biometrics and Information Sciences, AstraZeneca, One MedImmune Way, Gaithersburg, MD 20878 USA; 5 Global Medicines Development, AstraZeneca, One MedImmune Way, Gaithersburg, MD 20878 USA

**Keywords:** Asthma, IL-13, Predictive biomarker, SIDES (subgroup identification based on differential effect search), STRATOS 1, STRATOS 2, Subgroup identification, Tralokinumab (up to 10)

## Abstract

**Background:**

Tralokinumab is an anti–interleukin (IL)-13 monoclonal antibody investigated for the treatment of severe, uncontrolled asthma in two Phase III clinical trials, STRATOS 1 and 2. The STRATOS 1 biomarker analysis plan was developed to identify biomarker(s) indicative of IL-13 activation likely to predict tralokinumab efficacy and define a population in which there was an enhanced treatment effect; this defined population was then tested in STRATOS 2.

**Methods:**

The biomarkers considered were blood eosinophil counts, fractional exhaled nitric oxide (FeNO), serum dipeptidyl peptidase-4, serum periostin and total serum immunoglobulin E. Tralokinumab efficacy was measured as the reduction in annualised asthma exacerbation rate (AAER) compared with placebo (primary endpoint measure of STRATOS 1 and 2). The biomarker analysis plan included negative binomial and generalised additive models, and the Subgroup Identification based on Differential Effect Search (SIDES) algorithm, supported by robustness and sensitivity checks. Effects on the key secondary endpoints of STRATOS 1 and 2, which included changes from baseline in standard measures of asthma outcomes, were also investigated. Prior to the STRATOS 1 read-out, numerous simulations of the methodology were performed with hypothetical data.

**Results:**

FeNO and periostin were identified as the only biomarkers potentially predictive of treatment effect, with cut-offs chosen by the SIDES algorithm of > 32.3 ppb and > 27.4 ng/ml, respectively. The FeNO > 32.3 ppb subgroup was associated with greater AAER reductions and improvements in key secondary endpoints compared with the periostin > 27.4 ng/ml subgroup. Upon further evaluation of AAER reductions at different FeNO cut-offs, ≥37 ppb was chosen as the best cut-off for predicting tralokinumab efficacy.

**Discussion:**

A rigorous statistical approach incorporating multiple methods was used to investigate the predictive properties of five potential biomarkers and to identify a participant subgroup that demonstrated an enhanced tralokinumab treatment effect. Using STRATOS 1 data, our analyses identified FeNO at a cut-off of ≥37 ppb as the best assessed biomarker for predicting enhanced treatment effect to be tested in STRATOS 2. Our findings were inconclusive, which reflects the complexity of subgroup identification in the severe asthma population.

**Trial registration:**

STRATOS 1 and 2 are registered on ClinicalTrials.gov (NCT02161757 registered on June 12, 2014, and NCT02194699 registered on July 18, 2014).

**Electronic supplementary material:**

The online version of this article (10.1186/s12890-019-0889-4) contains supplementary material, which is available to authorized users.

## Background

Interleukin (IL)-13 is a type-2 pleiotropic cytokine thought to play a central role in asthma pathophysiology [1]. Overexpression of pulmonary IL-13 in transgenic mice led to development of features typical of asthma, such as eosinophilic airway inflammation, increased mucus production, sub-epithelial fibrosis and airway hyper-responsiveness [2]. In addition, in mice sensitised to ovalbumin, neutralisation of IL-13 resulted in the attenuation of airway hyper-responsiveness, goblet cell metaplasia and lung eosinophilia [3, 4]. Clinical data have demonstrated that people with atopic and non-atopic asthma have increased concentrations of IL-13 mRNA and IL-13 in sputum samples and bronchial biopsies compared with those without asthma [5–9].

The presumed role of IL-13 in asthma led to the clinical development of anti–IL-13 treatment strategies such as tralokinumab, an immunoglobulin (Ig) G_4_ human monoclonal antibody (mAb) that potently and specifically neutralises IL-13 by preventing its interaction with the IL-13 receptor α1 and α2 subunits [10, 11]. A Phase IIa tralokinumab trial in participants with moderate-to-severe uncontrolled asthma showed no improvement in asthma control in the all-comers population, but did show increases in forced expiratory volume in 1 s (FEV_1_). Analysis of participants by IL-13 axis activation revealed better outcomes with tralokinumab in those participants with IL-13 activation (sputum IL-13 ≥ 10 pg/ml) compared with participants with low or no activation (sputum IL-13 < 10 pg/ml), or those receiving placebo [12]. In a follow-up Phase IIb trial in a similar population, tralokinumab did not reduce the annualised asthma exacerbation rate (AAER) in the all-comers population. However, post-hoc analyses indicated enhanced benefits in participants with evidence of IL-13 axis activation, assessed by elevated serum concentrations of periostin or dipeptidyl peptidase-4 (DPP-4), which are biomarkers induced by IL-13 [13]. The data from these two Phase II trials suggested that tralokinumab would only be effective in severe asthma when there was evidence of IL-13 activation. This concept was supported by data from clinical trials of another anti–IL-13 mAb, lebrikizumab [14, 15]. It was also consistent with emerging evidence that underlying patterns of airway inflammation, and thus response to treatment, vary among people with severe asthma [16].

**Fig. 1:**
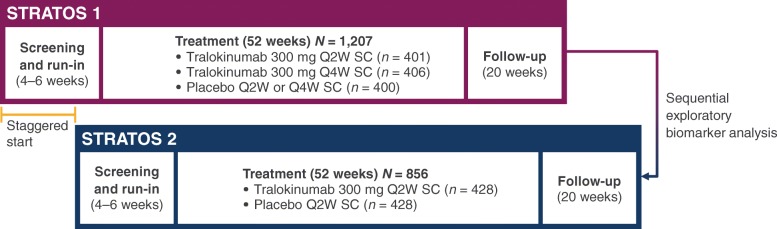
Staggered trial design of STRATOS 1 and 2. *Q2W*, every 2 weeks; *Q4W*, every 4 weeks; *SC*, subcutaneous

The tralokinumab late-stage clinical development programme in severe, uncontrolled asthma [17] was specifically designed to include two similar pivotal Phase III trials, STRATOS 1 (NCT02161757) and STRATOS 2 (NCT02194699), which were conducted in parallel but with staggered analyses (Fig. 1) [18]. STRATOS 1 primarily investigated the efficacy of tralokinumab in an all-comers population and, using an exploratory biomarker analysis plan, investigated several biomarkers that were potentially predictive of tralokinumab efficacy. The candidate biomarkers considered were blood eosinophil counts, fractional exhaled nitric oxide (FeNO), serum DPP-4 concentration, serum periostin concentration and total serum IgE concentration. These biomarkers are all continuous in nature and are either associated with IL-13 activation [19, 20] or with previous successful treatment of asthma with a biologic therapy [17, 21]. IL-13 was not assessed as a biomarker as circulating levels are very low, and when this study was conducted, available immunoassays did not reliably detect this protein [22].

The biomarker analysis results of STRATOS 1 were used for two purposes: to determine whether any of the biomarkers predicted a greater benefit with tralokinumab treatment; and to identify the threshold value for any predictive biomarker that would distinguish subgroups of participants with an enhanced benefit. These findings were then tested in STRATOS 2. Here, we describe the analyses applied in the identification of the best biomarker candidate and threshold value determined from STRATOS 1. The results of the primary analyses of STRATOS 1 and 2 in the biomarker-identified subgroup of participants has been published separately [23].

## Methods and results

### The STRATOS 1 and STRATOS 2 clinical trials

STRATOS 1 and 2 were both multicentre, randomised, double-blind, parallel-group, placebo-controlled Phase III clinical trials (Fig. 1). The two trials were conducted during an overlapping period, with the start and end dates staggered to allow for sequential analysis. They each had a 4–6-week run-in period, a 52-week treatment period and follow-up visits at Weeks 56 and 72 [18, 23]. In STRATOS 1, 1,207 participants were randomised 2:1:2:1 to receive either 300 mg tralokinumab or placebo subcutaneously (SC) every 2 weeks (Q2W), or 300 mg tralokinumab or placebo SC every 4 weeks (Q4W). In STRATOS 2, 856 participants were randomised 1:1 to receive either 300 mg tralokinumab or placebo SC Q2W [23].

The primary objective of STRATOS 1 was to investigate the effect of tralokinumab Q2W on the AAER up to Week 52 compared with placebo in an unselected all-comers population. The STRATOS 2 primary objective was originally to evaluate the effect of tralokinumab on the AAER in both an all-comers and a biomarker-positive population, but was amended to investigate only the biomarker-positive population as defined by the biomarker analysis of STRATOS 1. The analysis of the all-comers population was redefined as a secondary objective. Key secondary measures for STRATOS 1 and 2 were percentage change from baseline to Week 52 in pre-bronchodilator FEV_1_ and absolute change from baseline to Week 52 in scores of Asthma Control Questionnaire 6-item version (ACQ-6), Standardised Asthma Quality of Life Questionnaire for 12 years and older (AQLQ) and Asthma Symptom Score [18, 23].

### The biomarker analysis plan for STRATOS 1

The biomarker analysis plan for STRATOS 1 had two objectives:

1. To assess the relationship between continuous baseline values for the five identified biomarkers, AAER and treatment as the basis for identifying the biomarker with potential properties to predict the treatment effect of tralokinumab.
2. To determine the most appropriate threshold for the biomarker identified as having the best potential predictive properties of enhanced treatment effect.

The biomarker analyses of STRATOS 1 were based on the Full Analysis Set (FAS), defined as all randomised participants (irrespective of baseline biomarker concentration, the ‘all-comers’) who received any investigational product, regardless of protocol adherence and/or premature investigational product discontinuation or delay. The definition of the biomarker-positive subgroup, participants in the FAS with baseline biomarker concentrations equal to or greater than the identified threshold cut-off, was determined prior to unblinding of STRATOS 2. These biomarker analyses were focused on comparing the tralokinumab Q2W data with placebo data. For this purpose, the Q2W and Q4W placebo arms, which were well balanced in terms of demographic characteristics such as age, sex, race and ethnicity and had comparable lung function at baseline, were combined. Results for the tralokinumab Q4W arm (vs. combined placebo data) were used to support the Q2W findings. All analyses and the covariates used in each model were pre-specified in the STRATOS 1 statistical analysis plan, which was shared with the FDA. Analyses were conducted using either SAS software (version 9.4; SAS Institute Inc., Cary, NC) or R (version 3.2.4 [https://www.r-project.org/]).

The STRATOS 1 biomarker analysis plan was based on an understanding that a single statistical analysis would not be suitable for addressing the two objectives, instead requiring multiple approaches. Consequently, the plan was developed using various statistical approaches to answer four separate questions, as described in detail below. Once the biomarker analysis plan was developed, multiple realistic scenarios with different effect sizes and biomarker interactions were simulated in order to assess whether the full statistical methodology was able to detect known predictive signals, as well as to refine our ability to interpret the results and practice the decision-making process. These blinded scenario simulations were carried out prior to the read-out of STRATOS 1 by a statistician who was not otherwise involved in the analysis. The simulated data were based on modelled relationships between biomarkers, other covariates and exacerbations, developed using baseline data from STRATOS 1. All of the analyses outlined below were run using the simulated data and interpreted by the blinded clinical team, the results of which were used to improve the decision-making process for moving forward with particular biomarkers and subgroups. The scenarios considered potential differences in placebo rate, all-comers effect (i.e. the overall treatment effect) and various relationships between biomarkers and exacerbations. These simulation exercises confirmed the ability of the methodology to support adequate identification of biomarker-positive subgroups and, in turn, helped to overcome the difficulties in interpreting the results.

### Question 1: are baseline values of the five biomarkers predictive of treatment effect?

Before the predictive properties of the five candidate biomarkers were assessed, their distribution within the STRATOS 1 population and relationship with known potential risk factors for asthma exacerbations were assessed using descriptive statistics (Table 1). These potential risk factors included geographical region, number of exacerbations in the year prior to trial entry, body mass index, smoking status, inhaled corticosteroid (ICS) dosage, sex and age. Baseline concentrations of candidate biomarkers were similar across the treatment groups, but median concentrations of blood eosinophils (≈200 cells/μl) and FeNO (≈20.3 ppb) were relatively low for a severe asthma population. Clear relationships were found between biomarker concentrations and some non-biomarker covariates. Greater baseline FeNO, eosinophil and, to some degree, periostin concentrations were associated with a greater number of previous exacerbations, suggesting that these biomarkers were prognostic to some extent (Additional file 1: Figure S1). Greater baseline periostin concentrations were found in participants from the Asia/Pacific region compared with other regions, while greater baseline periostin and DPP-4 concentrations were observed in adolescents (Additional file 1: Figures S2 and S3).

**Table 1:** Distribution of baseline biomarker concentrations by treatment group in the STRATOS 1 all-comers population (full analysis set)

Biomarker		Combined placebo^a^(*N* = 400)	Tralo 300 mg Q2W(*N* = 398)	Tralo 300 mg Q4W(*N* = 404)
FeNO, ppb	n	398	395	397
	Mean (SD)	29.6 (28.2)	30.5 (30.6)	29.2 (29.0)
	Median (range)	20.2 (2.3–189.9)	20.3 (0.0–244.0)	20.4 (0.0–201.8)
Periostin, ng/ml	n	398	397	403
	Mean (SD)	25.4 (11.8)	26.3 (12.7)	26.0 (11.1)
	Median (range)	22.8 (7.6–83.4)	23.0 (9.5–91.6)	23.3 (8.7–78.8)
Eosinophils, cells/μl	n	393	393	395
	Mean (SD)	254 (204)	308 (468)	296 (381)
	Median (range)	200 (20–2,020)	210 (0–7,510)	200 (0–5,880)
DPP-4, ng/ml	n	399	397	404
	Mean (SD)	262.0 (75.6)	264.0 (91.8)	267.0 (77.0)
	Median (range)	251.0 (92.0–617.0)	246.0 (87.0–766.0)	254.0 (103.0–721.0)
IgE, IU/ml	n	395	392	399
	Mean (SD)	432.0 (786.2)	429.2 (929.0)	440.4 (974.2)
	Median (range)	165.9 (2.1–5,347)	141.8 (1.4–7,580)	141.8 (0.5–8,423)

*DPP-4* Dipeptidyl peptidase-4, *FeNO* Fractional exhaled nitric oxide; *IgE* Immunoglobulin E, *IU* International units, *SD* Standard deviation, *Q2W* Every 2 weeks, *Q4W* Every 4 weeks, *Tralo* Tralokinumab

^a^The placebo treatment group is a pooled treatment group (placebo Q2W + placebo Q4W)

**Fig. 2:**
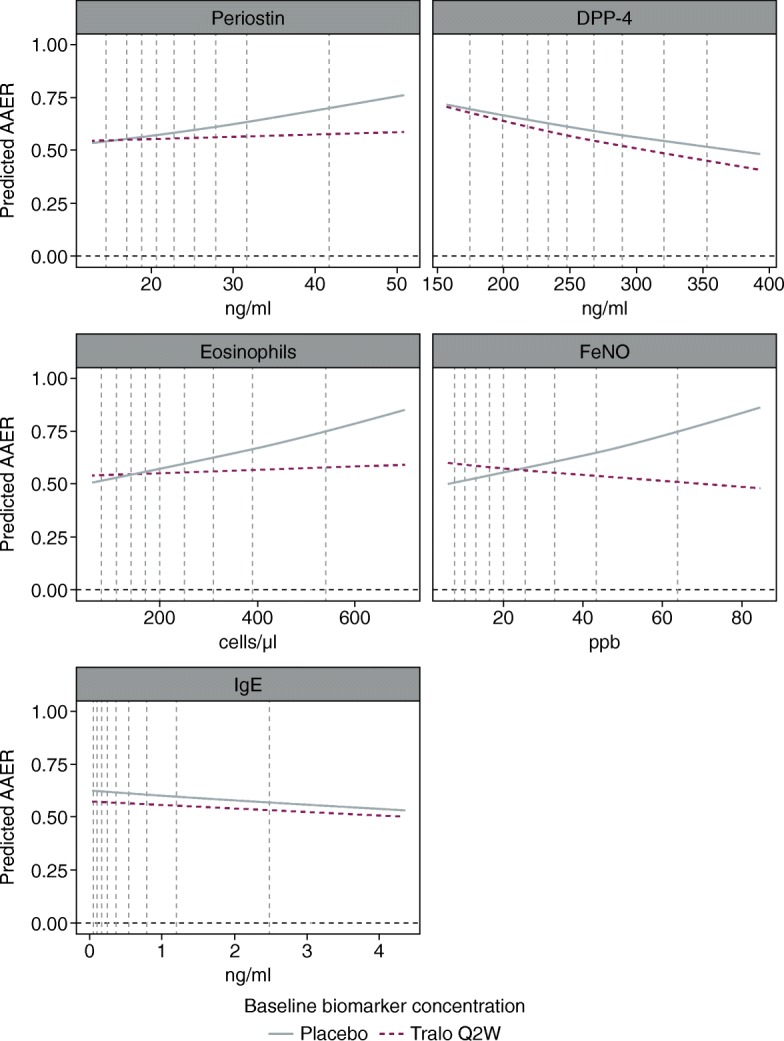
Estimated relationships between biomarkers and annualised asthma exacerbation rate, predicted using negative binomial models in the STRATOS 1 all-comers population (full analysis set)*. *Estimates were based on negative binomial models including treatment group, geographical region, age, number of exacerbations in the previous year, biomarker and treatment*biomarker as covariates. The log of each participants’s corresponding follow-up time was used as an offset variable in the model to adjust for participants having different exposure times during which asthma exacerbations occurred. The two placebo groups were pooled before the analyses. Predictions for biomarker values between the 5 to 95% quantiles for each biomarker are shown, but all data are used in the estimation. Vertical dashed lines show the 10th to 90th percentiles. Two participants with outlier eosinophil values (7,510 and 4,130 cells/μl) were not included in the analyses. *AAER*, annual asthma exacerbation rate; *DPP-4*, dipeptidyl peptidase-4; *FeNO*, fractional exhaled nitric oxide; *IgE*, immunoglobulin E; *Q2W*, every 2 weeks; *Tralo*, tralokinumab

To investigate the potential biomarker predictive properties of the five biomarkers expressed as continuous variables, graphs were created to present the relationship between AAER and baseline biomarker concentration (Fig. 2). For these graphs, negative binomial models were used to assess treatment effect (measured as AAER) with covariates of treatment group, geographical region, age and number of exacerbations in the previous year. The log of each participant’s corresponding follow-up time was used in the models as an offset variable to adjust for participants having different exposure times during which asthma exacerbations occurred. These graphs demonstrated greater exacerbation rates in the placebo group with increasing baseline concentrations of FeNO, periostin and eosinophils, suggesting a prognostic relationship. They also showed that the exacerbation rate did not increase with greater baseline concentrations of these biomarkers in the tralokinumab treatment group, suggesting a predictive relationship.

**Fig. 3:**
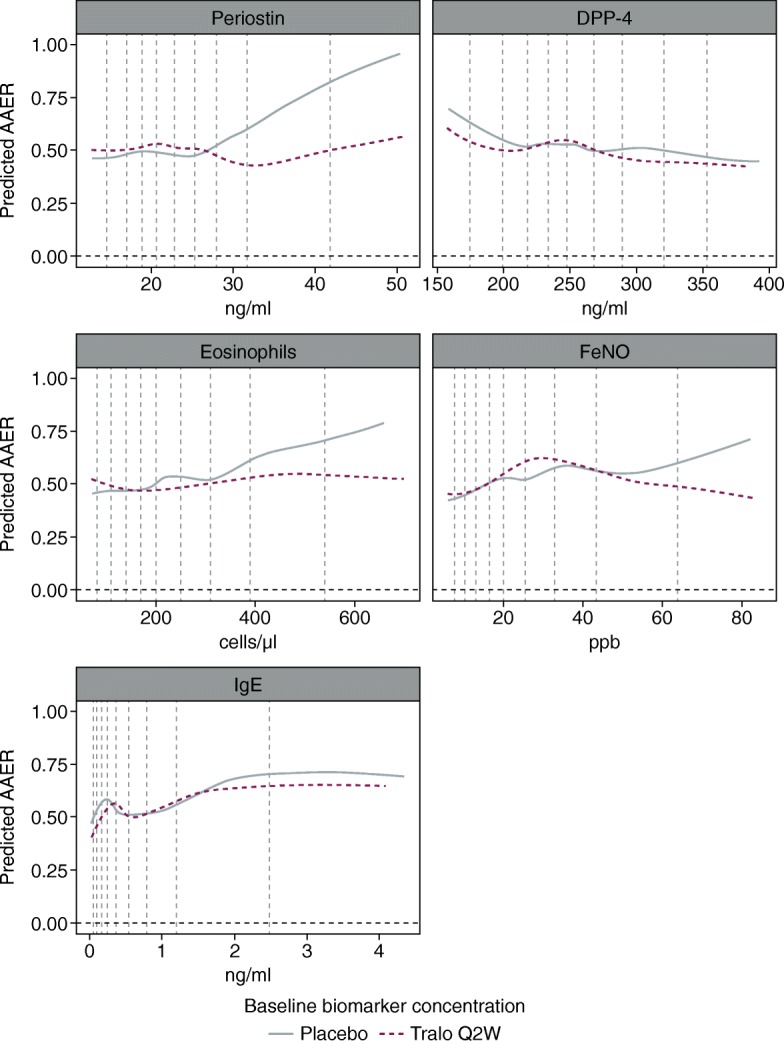
Estimated relationships between biomarkers and annualised asthma exacerbation rate, predicted using generalised additive models in the STRATOS 1 all-comers population (full analysis set)*. *Estimates were based on negative binomial models including treatment group, geographical region, age, number of exacerbations in the previous year and s(biomarker, by treatment) as covariates. Smoothing splines (s) are fitted by penalised likelihood using thin plate regression splines (mgcv R package). The log of each participants’s corresponding follow-up time was used as an offset variable in the model to adjust for participants having different exposure times during which asthma exacerbations occurred. Graphs show the exp.(LOESS[predicted GAM link function for each participant]), where 0.67 is the span used in the LOESS. Only data between the 5 to 95% quantiles for each biomarker are shown, but all data are used in the estimation. The two placebo groups were pooled before the models were estimated. Vertical dashed lines show the 10th to 90th percentiles. Two participants with outlier eosinophil values (7,510 and 4,130 cells/μl) were not included in the analyses. *AAER*, annual asthma exacerbation rate; *DPP-4*, dipeptidyl peptidase-4; *FeNO*, fractional exhaled nitric oxide; *GAM*, generalised additive models; *IgE*, Immunoglobulin E; *LOESS*, local polynomial regression; *Q2W*, every 2 weeks, *Tralo*, tralokinumab

The biomarker predictive properties were investigated further using Generalized Additive Models (GAM), a type of generalised linear model, with smoothing splines used to visualise potential relationships. In GAM, some of the (log-)linear X terms are replaced with a fitted smooth function, f(X), to give a visual representation of the shape of f(biomarker) and therefore potential relationships in the data. The plots produced using GAM visually supported the predictive and prognostic properties of baseline concentrations of FeNO, periostin and, to a lesser extent, eosinophils (Fig. 3).

Likelihood ratio tests were used to provisionally quantify the predictive properties of the continuous biomarkers by assessing the impact of biomarker-by-treatment interactions. Firstly, a negative binomial model that included all interaction terms for all five candidate biomarkers versus treatment was compared with a model without interaction terms. Secondly, separate models with and without treatment-by-biomarker interaction terms were compared for each individual candidate biomarker. These assessments provided exploratory interaction effects for each biomarker, but had low power and were only able to identify non-complex linear relationships. Noting these limitations, these analyses found nominally significant (*p* < 0.10) interaction effects for FeNO in both tralokinumab treatment groups and for periostin in the Q4W, but not the Q2W, group (Table 2).

**Table 2:** Assessment of interaction between biomarkers and treatment effect in the STRATOS 1 all-comers population (full analysis set)^a^

Biomarker	Tralo Q2W *p*-value^†^	Tralo Q4W *p*-value^†^
All (likelihood ratio test)	0.305	0.132
FeNO	0.038	0.086
Periostin	0.478	0.090
Eosinophils	0.176	0.395
DPP-4	0.695	0.848
IgE	0.946	0.297

*DPP-4* Dipeptidyl peptidase, *FeNO* Fractional exhaled nitric oxide, *IgE* Immunoglobulin E, *Q2W* Every 2 weeks, *Q4W* Every 4 weeks, *Tralo* Tralokinumab

^a^Likelihood ratio tests have low power and are only able to identify linear relationships

^†^*p*-values were calculated using negative binomial models including treatment group, geographical region, age, number of exacerbations in the previous year, biomarker and treatment*biomarker as covariates. The log of each participant’s corresponding follow-up time was used as an offset variable in the model to adjust for participants having different exposure times during which asthma exacerbations occurred. *p*-values for each individual biomarker represents the Wald statistic for a biomarker*treatment interaction term using separate models. The test of all five biomarkers was based on a likelihood ratio test comparing a model including the baseline covariates above, biomarker and treatment interaction terms for all five biomarkers with a model including terms for the baseline covariates and biomarkers but not any treatment interaction terms

**Fig. 4:**
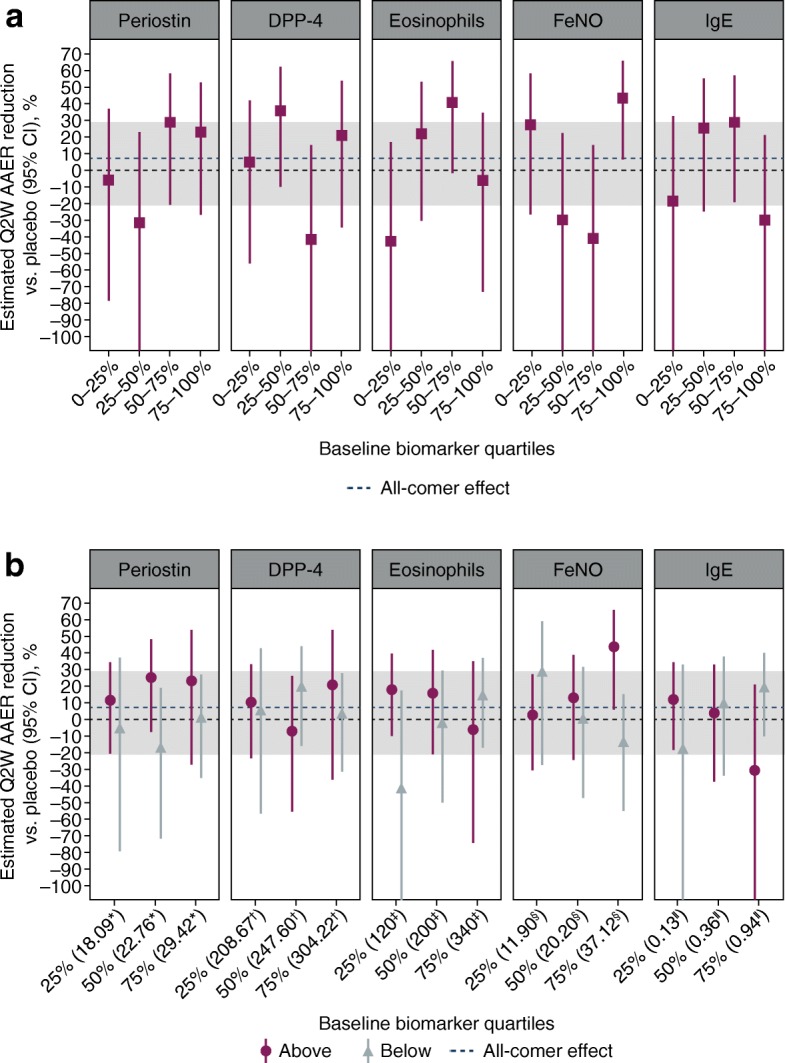
Treatment effect with tralokinumab Q2W* within biomarker quartile groups (**a**), and in biomarker-high and -low subgroups defined by cumulative cut-offs (**b**) in the STRATOS 1 all-comers population (full analysis set). *Estimates within subgroups were based on negative binomial models including treatment group, geographical region, age, number of exacerbations in the previous year, biomarker group and treatment*biomarker group as covariates. The log of each participants’s corresponding follow-up time was used as an offset variable in the model to adjust for participants having different exposure times during which asthma exacerbations occurred. ‘Above’ and ‘below’ in panel B refer to participants with baseline biomarker concentrations falling above and below the indicated cut-off, respectively. The lower CI limits are truncated at − 110%. Two participants with outlier eosinophil values (7,510 and 4,130 cells/μl) were not included in the analyses. The two placebo groups were pooled before the analyses. ^†^ng/ml. ^‡^Cells/μl. ^§^ppb. *AAER*, annualised asthma exacerbation rate; *CI*, confidence interval; *DPP-4*, dipeptidyl peptidase-4; *FeNO*, fractional exhaled nitric oxide; *IgE*, immunoglobulin E; *Q2W*, every 2 weeks

In addition to investigating the biomarkers as continuous variables, Forest plots were used to show the AAER reduction with tralokinumab versus placebo for the five biomarkers both within each quartile group (Fig. 4a), and in biomarker-high and -low subgroups defined by cumulative cut-offs based on quartiles (Fig. 4b). These data were estimated using negative binomial models that, in addition to treatment group, biomarker group, treatment*biomarker group and time on study, included covariates with which the outcome was likely to correlate, such as geographical region, age and previous number of exacerbations in the past year; these covariates were also included in the model used for the primary analysis of STRATOS 2. Within-quartile grouping indicated that the treatment effect was greatest at high baseline concentrations of both FeNO and periostin (Fig. 4a), with a similar pattern demonstrated with increasing cut-offs of FeNO (Fig. 4b). This suggested that FeNO and periostin were potentially predictive of treatment response to tralokinumab.

### Question 2: is the choice of a biomarker-positive subgroup defensible?

The collective evidence from the above analyses identified FeNO and periostin as biomarkers that were prognostic and potentially predictive of response to tralokinumab. The Subgroup Identification based on Differential Effect Search (SIDES) algorithm [24, 25] was used to further support the predictive properties of these candidate biomarkers and to identify the respective cut-off values for an enhanced response to tralokinumab.

**Fig. 5:**
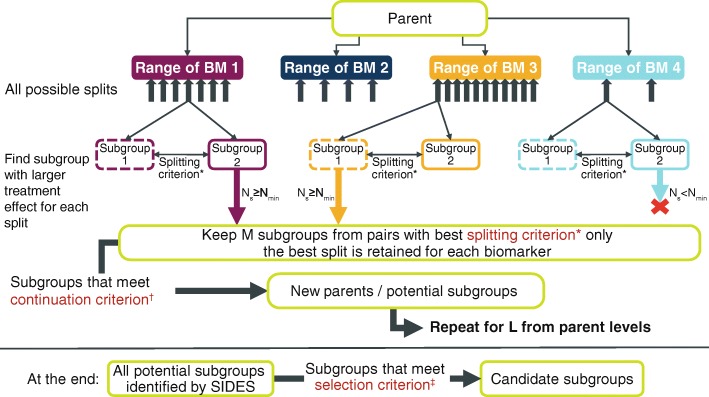
Overview of the SIDES algorithm. *The splitting criterion is used to determine which child subgroups have improved efficacy and either comparable or improved safety compared with other child subgroups; for each biomarker, only the best split according to the splitting criterion is considered in the next step. There are four types of splitting criteria, one of which is applied to each SIDES run [24]: Criterion 1: maximising the differential effect between the two child subgroups. Criterion 2: maximising the treatment effect in at least one of the two child subgroups. Criterion 3: criterion 3 is a combination of criteria 1 and 2; it is used if criterion 1 is met (i.e. a difference is identified and the *p-*value is significant), but criterion 2 is not (the treatment effect in either subgroup is not significant). Criterion 4: maximising the differential effect between the two child subgroups in terms of both efficacy and safety. ^†^The continuation criterion aims to reduce the number of child subgroups tested by only pursuing those that demonstrate improvements compared with their parent [24]. ^‡^The selection criterion is used to screen subgroups to identify only those in which the treatment effect reaches a threshold of clinical relevance [24]. *BM*, biomarker; *L*, maximum number of covariates defining a subgroup; *M*, maximum number of best candidate covariates to be considered at each step to define child subgroups; *N*_*s*_, size of the subgroup with largest treatment effect based on the split; *N*_*min*_, minimum allowed subgroup size

SIDES recursively partitions specific areas of the covariate space associated with treatment benefit using a treatment effect–based splitting criterion in order to identify the best split for each covariate [24, 25] (Fig. 5). In the search algorithm, a negative binomial model was used to estimate the treatment effect, which included treatment group as a covariate, as well as the log of each participant’s corresponding follow-up time as an offset variable. When further assessing the identified subgroups, age, geographical region and number of previous exacerbations were included as covariates to match the model that was to be used in the primary analysis of STRATOS 2. Because AAER reduction, the primary outcome measure of tralokinumab treatment effect, was a count variable and the SIDES package available at the time of these analyses did not allow for the modelling of count data via negative binomial models, a bespoke package was developed in collaboration with I Lipkovich for the analysis of STRATOS 1 and 2 (it should be noted that the latest version of SIDES allows for count data modelling).

**Fig. 6:**
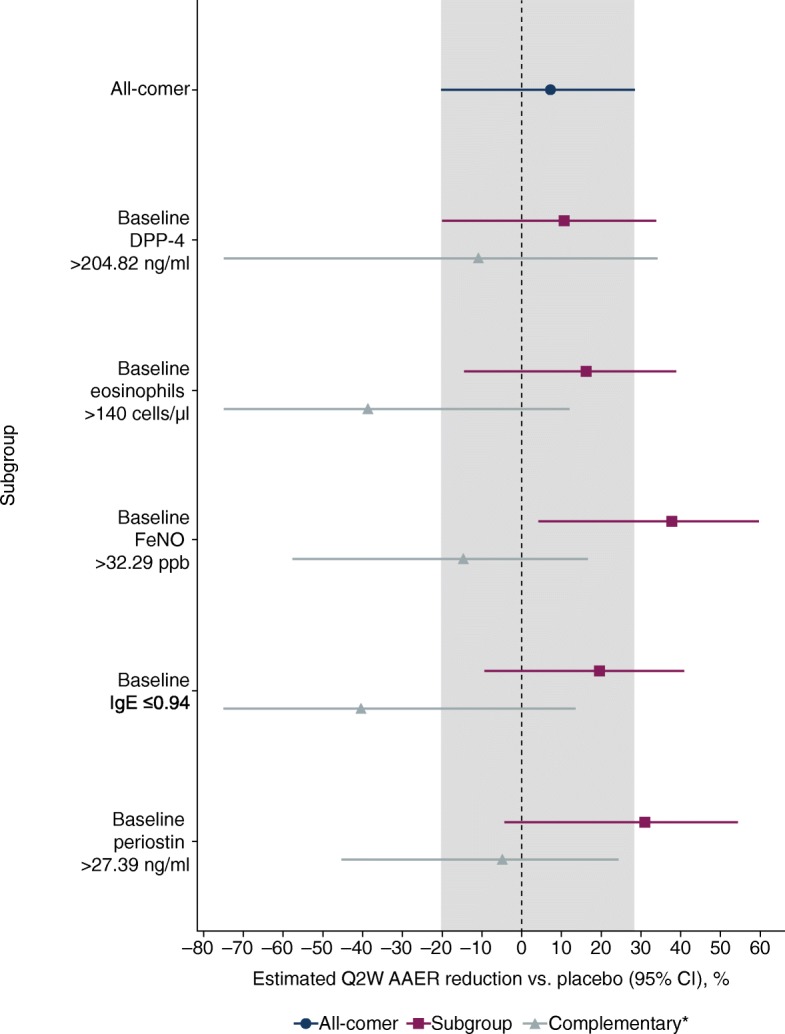
SIDES subgroups and tralokinumab treatment effect in the STRATOS 1 all-comers population (full analysis set)*. *Estimates within subgroups were based on negative binomial models including treatment group, geographical region, age and number of exacerbations in the previous year. The log of each participants’s corresponding follow-up time was used as an offset variable in the model to adjust for participants having different exposure times during which asthma exacerbations occurred. The lower CI limits truncated at − 75%. Results are shown for the tralokinumab Q2W and pooled placebo arms based on a negative binomial model adjusted for baseline covariates. ^†^The placebo treatment group is a pooled treatment group (placebo Q2W + placebo Q4W). ^‡^Complementary refers to the subgroup of participants not in the subgroup of interest, i.e. participants with baseline biomarker concentrations of: DPP-4 ≤ 204.8 ng/ml; eosinophils ≤140 cells/μl; FeNO ≤32.3 ppb; IgE > 0.9 ng/ml; periostin ≤27.4 ng/ml. *AAER*, annualised asthma exacerbation rate; *CI*, confidence interval; *DPP-4*, dipeptidyl peptidase-4; *FeNO*, fractional exhaled nitric oxide; *IgE*, immunoglobulin E; *Q2W*, every 2 weeks; *SIDES*, Subgroup Identification based on Differential Effect Search

In order to restrict how complex the subgroups identified by SIDES could be, it was pre-specified that the prevalence of a biomarker cut-off was required to be at least 30% in the study population and subgroups could only be based on one of the candidate biomarkers. Additional SIDES analyses were conducted in which subgroups could be identified using either multiple biomarkers or non-biomarker covariates to aid understanding of how the biomarkers influenced the effect of tralokinumab. The results of the SIDES analysis were presented in Forest plots, which confirmed the potential predictive properties of FeNO and periostin and identified baseline cut-off values of > 32.3 ppb and > 27.4 ng/ml, respectively (Fig. 6). The FeNO biomarker subgroup identified through SIDES had a slightly better AAER reduction with tralokinumab versus placebo than the periostin subgroup (38% versus 31%).

**Fig. 7:**
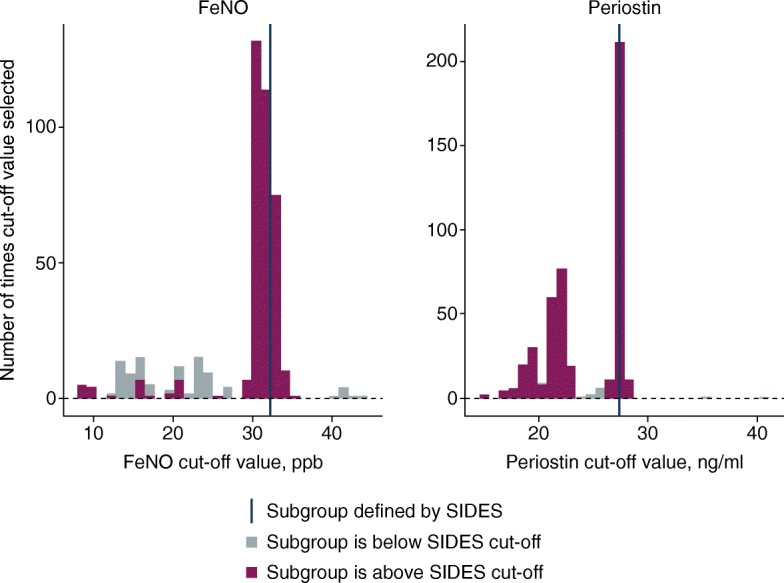
Uncertainty in biomarker cut-offs in subgroups identified by SIDES in the STRATOS 1 all-comers population (full analysis set)*. *Number of bootstrap samples was 500. *FeNO*, fractional exhaled nitric oxide; *SIDES*, Subgroup Identification based on Differential Effect Search

The SIDES-identified biomarker cut-off values were confirmed using robustness and sensitivity analyses; for example, assessing the effects of minor parameter modifications and removing the restriction on subgroup size (see Additional file 2 for further details). The certainty of the identified cut-offs was tested by bootstrapping the data (i.e. a number of bootstrap sample populations were created by sampling with replacement data from the STRATOS 1 study) and then re-running SIDES on each bootstrap sample, resulting in a range of cut-offs (across 50 evenly distributed splits) for each biomarker. The resulting plots identified how many times the cut-off values were chosen for each biomarker and whether the subgroups chosen (with greater observed efficacy) were above or below the cut-off value. These results were then compared with the ‘best’ cut-off identified by the initial SIDES analysis. The comparison showed a greater degree of certainty (i.e. less variability) with FeNO than with periostin (Fig. 7), which while exploratory, may reflect the uncertainty in our understanding of the roles of various cytokines and biomarkers in the pathophysiology of severe asthma.

**Fig. 8:**
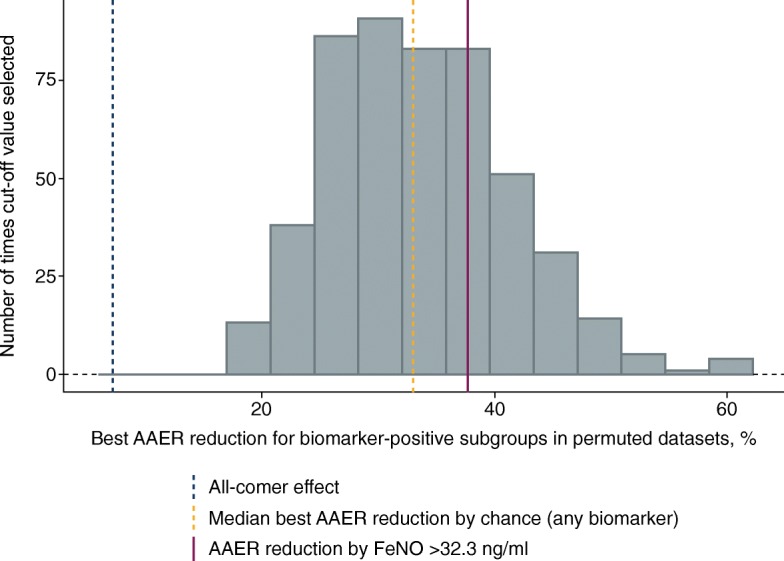
Result of SIDES on permuted data in the STRATOS 1 all-comers population (full analysis set)*. *The plot provides an indication of how SIDES would perform on these data if there was no predictive biomarker. It is computed by permuting the five biomarkers, but removing all predictive (and prognostic) biomarker effects and re-running SIDES. Number of permutations was 500. *AAER*, annualised asthma exacerbation rate; *FeNO*, fractional exhaled nitric oxide; *SIDES*, Subgroup Identification based on Differential Effect Search

A permutation approach was used to assess the likelihood of recording the observed AAER reduction by chance in any subgroup when no true connection between biomarker and treatment existed. The biomarker variables were randomly permuted against participant-level data (treatment, exacerbation history, etc.) to remove the biomarker effects and leave only the overall treatment effect. These data were then run through SIDES to identify the subgroup (defined by any of the five candidate biomarkers) with the best treatment effect. The process was repeated 500 times to give a distribution of permuted ‘best subgroup’ effects by chance that were then compared with the results obtained in the initial SIDES analysis for each biomarker (Fig. 8). Based on this analysis, the median best AAER reduction observed by chance was estimated to be 33%. The effect observed in the main SIDES analysis for the FeNO subgroup (38% reduction) was greater than this value, although still within the distribution of ‘chance’ results; in contrast, the effect observed in the periostin subgroup (31% reduction) was slightly lower. This analysis provided support for choosing FeNO over periostin as the biomarker to assess further.

Alongside the evaluation of the secondary endpoints in the biomarker subgroups (described below), further analyses were conducted to assess the treatment effect on AAER in subgroups defined by FeNO cut-off values ranging from 30 to 40 ppb (Table 3). This was done using a negative binomial model with treatment group, geographical region, age, number of exacerbations in the previous year, treatment*biomarker group interaction and periostin group at baseline as covariates. Based on this analysis, a threshold of FeNO ≥37 ppb provided the best AAER reduction with tralokinumab treatment. Similar further analyses were not conducted for periostin following the assessment of the key secondary endpoints (described below) using the cut-off value identified by SIDES (> 27.4 ng/ml).

**Table 3:** Analysis of annualised asthma exacerbation rate reduction by baseline FeNO concentration in the STRATOS 1 all-comers population (full analysis set)^a^

FeNO cut-off, ppb	Tralo Q2W vs. combined placebo^b^, n	Rate ratio (95% CI)	*p*-value
≥30	133 vs. 135	0.71 (0.46, 1.10)	0.121
< 30	262 vs. 263	1.10 (0.79, 1.54)	0.559
≥31	128 vs. 127	0.69 (0.44, 1.07)	0.098
< 31	267 vs. 271	1.11 (0.80, 1.54)	0.533
≥32	124 vs. 124	0.67 (0.43, 1.05)	0.078
< 32	271 vs. 274	1.12 (0.81, 1.56)	0.482
≥33	118 vs. 122	0.64 (0.40, 1.02)	0.058
< 33	277 vs. 276	1.13 (0.82, 1.55)	0.460
≥34	113 vs. 114	0.64 (0.40, 1.03)	0.066
< 34	282 vs. 284	1.12 (0.81, 1.53)	0.496
≥35	105 vs. 108	0.62 (0.38, 1.01)	0.056
< 35	290 vs. 290	1.12 (0.82, 1.53)	0.478
≥36	103 vs. 106	0.61 (0.37, 1.01)	0.052
< 36	292 vs. 292	1.12 (0.82, 1.52)	0.488
≥37	97 vs. 102	0.56 (0.34, 0.94)	**0.028**
< 37	298 vs. 296	1.14 (0.84, 1.56)	0.391
≥38	94 vs. 98	0.58 (0.35, 0.98)	**0.040**
< 38	301 vs. 300	1.12 (0.82, 1.52)	0.468
≥39	92 vs. 92	0.66 (0.38, 1.12)	0.122
< 39	303 vs. 306	1.04 (0.77, 1.41)	0.789
≥40	88 vs. 89	0.67 (0.39, 1.16)	0.151
< 40	307 vs. 309	1.03 (0.76, 1.39)	0.847

*CI* Confidence interval, *FeNO* Fractional exhaled nitric oxide, *Q2W* Every 2 weeks, *Q4W* Every 4 weeks, *Tralo* Tralokinumab

^a^Rate ratios and *p*-values are from a negative binomial model analysis, with treatment group, geographical region, age group, periostin group at baseline, number of exacerbations in the previous year and treatment^*^biomarker group included as covariates. The log of each participant’s corresponding follow-up time was used as an offset variable in the model to adjust for participants having different exposure times during which asthma exacerbations occurred. Total follow-up time was defined as the time from randomisation to Week 52 or last contact if the participant was lost to follow-up. No multiplicity adjustments were made and all *p*-values stated are nominal; values <0.05 are bolded

^b^The placebo treatment group is a combined treatment group (placebo Q2W + placebo Q4W)

### Question 3: is there consistency of predictive effect across key secondary efficacy endpoints?

To evaluate further the choice of biomarker and threshold, key secondary endpoints in STRATOS 1 (percentage change from baseline in FEV_1_, and absolute changes from baseline in ACQ-6 score, AQLQ score and Asthma Symptom Score) were analysed using repeated measures models for the FeNO- and periostin-defined subgroups. Nominally significant improvements versus placebo in all key secondary endpoints – except for Asthma Symptom Score – were observed in the FeNO ≥37 ppb subgroup (Table 4); similar results were observed in the subgroup with FeNO ≥32.3 ppb (data not shown). In contrast, there was no consistent enhancement of treatment effect in the periostin > 27.4 ng/ml subgroup (Table 5).

**Table 4:** Analysis of key secondary endpoints by baseline FeNO concentration in the STRATOS 1 all-comers population (full analysis set)^a^

Change from baseline in	FeNO cut-off, ppb	Tralo Q2W vs. combined placebo^b^, n	Treatment effect (95% CI)	*p*-value
FEV_1_, %	≥37	87 vs. 92	12.80 (5.34, 20.26)	**< 0.001**
	< 37	268 vs. 269	3.83 (− 0.46, 8.12)	0.080
ACQ-6 score	≥37	75 vs. 83	−0.43 (− 0.71, − 0.16)	**0.002**
	< 37	242 vs. 231	− 0.07 (− 0.23, 0.09)	0.372
AQLQ score	≥37	74 vs. 83	0.53 (0.22, 0.84)	**< 0.001**
	< 37	241 vs. 230	0.02 (−0.16, 0.20)	0.860
Total Asthma Symptom Score	≥37	71 vs. 68	−0.05 (− 0.34, 0.24)	0.720
	< 37	242 vs. 243	−0.10 (− 0.26, 0.06)	0.222

*ACQ-6* Asthma Control Questionnaire (6-item), *AQLQ* Standardised Asthma Quality of Life Questionnaire for 12 Years and Older, *CI* Confidence interval, *FeNO* Fractional exhaled nitric oxide, *FEV*_*1*_ Forced expiratory volume in 1 s, *Q2W* Every 2 weeks, *Q4W* Every 4 weeks, *Tralo* Tralokinumab

^a^The two treatment arms were compared with the pooled placebo group using a repeated measures analysis. For percentage change in FEV_1_ the model was treatment group + geographical region + age group + periostin group at baseline + number of exacerbations in the previous year + visit + treatment*visit + treatment*biomarker group using an unstructured variance-covariance matrix. For change in score the model was baseline score + treatment group + geographical region + age group + periostin group at baseline + number of exacerbations in the previous year + visit + treatment*visit + treatment*biomarker group using a compound symmetric variance-covariance matrix. Baseline was the last non-missing measurement recorded prior to randomisation (typically randomisation). No multiplicity adjustments were made and all *p*-values stated are nominal; values <0.05 are bolded

^b^The placebo treatment group is a combined treatment group (placebo Q2W + placebo Q4W)

**Table 5:** Analysis of key secondary endpoints by baseline periostin concentration in the STRATOS 1 all-comers population (full analysis set)^a^

Change from baseline in	Periostin cut-off, ng/ml	Tralo Q2W vs. combined placebo^b^, n	Treatment effect (95% CI)	*p*-value
FEV_1_, %	> 27.4	117 vs. 120	6.61 (0.07, 13.14)	**0.048**
	≤27.4	239 vs. 241	5.54 (1.00, 10.09)	**0.017**
ACQ-6 score	> 27.4	104 vs. 103	− 0.11 (− 0.35, 0.13)	0.372
	≤27.4	212 vs. 211	−0.20 (− 0.37, − 0.03)	**0.022**
AQLQ score	> 27.4	104 vs. 103	0.09 (−0.18, 0.36)	0.529
	≤27.4	210 vs. 210	0.18 (−0.01, 0.37)	0.060
Total Asthma Symptom Score	> 27.4	103 vs. 96	0.03 (−0.22, 0.27)	0.843
	≤27.4	210 vs. 216	−0.15 (− 0.32, 0.03)	0.098

*ACQ-6* Asthma Control Questionnaire (6-item), *AQLQ* Standardised Asthma Quality of Life Questionnaire for 12 Years and Older, *CI* Confidence interval, *FEV*_*1*_ Forced expiratory volume in 1 s, *Q2W* Every 2 weeks, *Q4W* Every 4 weeks, *Tralo* Tralokinumab

^a^The two treatment arms were compared with the pooled placebo group using repeated measures analyses. For percentage change in FEV_1_ the model was treatment group + geographical region + age group + periostin group at baseline + number of exacerbations in the previous year + visit + treatment*visit + treatment*biomarker group using an unstructured variance-covariance matrix. For change in score the model was baseline score + treatment group + geographical region + age group + periostin group at baseline + number of exacerbations in the previous year + visit + treatment*visit + treatment*biomarker group using a compound symmetric variance-covariance matrix. Baseline was the last non-missing measurement recorded prior to randomisation (typically randomisation). No multiplicity adjustments were made and all *p*-values stated are nominal; values <0.05 are bolded

^b^The placebo treatment group is a combined treatment group (placebo Q2W + placebo Q4W)

The combined observations obtained through the above statistical methods supported the choice of FeNO as the preferred predictive biomarker with the threshold of FeNO ≥37 ppb. An overall comparison of the findings with FeNO ≥37 ppb and periostin > 27.4 ng/ml is shown in Table 6.

**Table 6:** Comparison of FeNO and periostin as a predictive biomarker in the STRATOS 1 all-comers population (full analysis set)

	FeNO ≥37 ppb	Periostin > 27.4 ng/ml
Prevalence in STRATOS 1, % (n/N)	23.7 (285/1,202)	32.9 (395/1,202)
Observed AAER reduction (95% CI) for tralo Q2W vs. combined placebo^a^, %	44 (6, 66)	31 (−4, 54)
Interaction test *p*-value^†^	**0.038**	0.478
Secondary endpoints enhanced for tralo Q2W	Consistent effect with FEV_1_, AQLQ, ACQ-6	No consistent effect observed
Observations for tralo Q4W	Consistent	Consistent effect only with the AAER reduction endpoint

*ACQ-6* Asthma Control Questionnaire (6-item), *AAER* Annualised asthma exacerbation rate, *AQLQ* Standardised Asthma Quality of Life Questionnaire for 12 Years and Older, *CI* Confidence interval, *FeNO* Fractional exhaled nitric oxide, *FEV*_*1*_ Forced expiratory volume in 1 s, *Q2W* Every 2 weeks, *Q4W* Every 4 weeks, *Tralo* Tralokinumab

^a^The placebo treatment group is a combined treatment group (placebo Q2W + placebo Q4W)

^†^*p*-value of 0.10 is considered nominally significant (depicted in bold)

### Question 4: is there a safety signal in the chosen biomarker subgroup?

Adverse event (AE) reporting for the subgroups of FeNO ≥37 ppb and FeNO < 37 ppb were evaluated. Reporting rates of overall AEs, serious AEs (SAEs) and AEs leading to study drug discontinuation were similar for the two subgroups. Reporting rates of individual AEs were also similar for the two subgroups (data reported elsewhere [23]).

## Discussion

Asthma is common, affecting around 339 million people worldwide [26]; up to 10% of these individuals have severe disease [27, 28]. People with severe asthma, despite established standards of care, experience diminished health-related quality of life, acute asthma exacerbations with frequent emergency room visits and hospitalisations, and thereby consume the majority of asthma-related healthcare resources [29–31]. There remains a significant unmet clinical need for these individuals, which has been partly met by the recent development of biologics. However, the five biologics currently approved for the treatment of asthma are not effective in all people with severe asthma, and rely on biomarkers to identify the individuals who are most likely to benefit from their use. These biomarkers are total serum IgE for omalizumab [32] and blood eosinophil counts for benralizumab [33], mepolizumab [34], reslizumab [35] and dupilumab [36]. The reason for the differences in the indicated patient profiles for these biologics is that asthma is a heterogeneous disease; there are different underlying mechanisms of airway inflammation driving disease and, thus, affecting treatment response [16]. The Phase II trials with tralokinumab illustrate this point. Effect was not found in the all-comer populations in these trials, but enhanced benefit was observed in subgroups of participants who had evidence of IL-13 axis activation [12, 13]. Unfortunately, the Phase II trials did not identify the biomarker with the best predictive properties for tralokinumab efficacy. The Phase III clinical development programme, which included the pivotal trials STRATOS 1 and 2, was therefore designed to first evaluate whether tralokinumab was effective in the all-comers severe asthma population, and second to determine whether there was a biomarker that identified a subgroup with an enhanced treatment benefit with tralokinumab [37].

We have presented the statistical methods employed in the biomarker analyses of the tralokinumab Phase III clinical trial, STRATOS 1. The aim of these analyses was to identify the biomarker and associated cut-off value most likely to define a biomarker-positive participant subgroup with an enhanced tralokinumab treatment effect. Based on the totality of evidence, FeNO with a cut-off of ≥37 ppb was considered the best option.

Biomarker-positive subgroup identification is extremely complex, with a wide variety of approaches available. As the number of biomarkers believed to predict tralokinumab treatment effect is small, machine learning methods that identify variables by relative influence, such as random forest [38], virtual twins [39] and gradient boosting models [40], were not appropriate. These methods primarily identify and rank potential biomarkers from a very large pool of candidates, but do not provide estimates of treatment effects or suggested cut-offs [41]. Bayesian model approaches were rejected as they are typically used for the analysis of pre-specified subgroups and also require the specification of a prior distribution, the choice of which can have a substantial impact on the result [42, 43]. Instead, we used a structured approach relying on multiple statistical methods. The introduction of the SIDES algorithm into this structured approach was useful for several reasons. The SIDES algorithm is reproducible and intuitive, with the advantage that the outputs are easily interpretable. Most importantly for our needs, it identifies potential predictive biomarkers while simultaneously determining the cut-off values for defining subgroups and allowing explicit control of subgroup complexity [24]. The search methodology of SIDES can incorporate covariate-adjusted estimates of treatment effect in subgroups and is less restrictive than tree-based algorithms, allowing evaluation of multiple overlapping subgroups [24]. Finally, as we have demonstrated here, the method can be easily adapted to new types of data. In comparison, classical methods such as interaction tests have low power, only measure linear contributions, would suffer from a greater degree of multiplicity and do not provide biomarker cut-off values [24]. This flexibility allowed for the exploration of various options and for an increased understanding of how biomarkers impact the treatment effect of tralokinumab.

Of the five biomarkers we tested, the two identified as most likely to predict enhanced tralokinumab treatment effect were FeNO and periostin. Concentrations of both of these biomarkers are directly related to IL-13 axis activation. High concentrations of FeNO are associated with elevated type-2 inflammation [44], an increased risk of asthma exacerbations [45, 46] and steroid insensitivity in people with asthma [47]. It is produced in the airways by inducible NO synthase [48], an enzyme that is upregulated by IL-13 [49]. FeNO has previously been investigated as a biomarker in clinical trials of biologics for the treatment of asthma, often as a surrogate biomarker of eosinophilic inflammation, and has demonstrated predictive properties for improved treatment responses [14, 50–54]. Whilst the best FeNO cut-off we identified using SIDES was > 32.3 ppb, upon further investigation of the tralokinumab effect on AAER reduction and secondary endpoints we established the subgroup defined by a cut-off of ≥37 ppb as the best choice. This was because – based on the dataset in hand – it predicted the greatest treatment effect with tralokinumab in STRATOS 1, despite the prevalence of participants with baseline FeNO ≥37 ppb in STRATOS 1 and 2 being lower than the minimum we had originally planned for (24% [285/1,202] and 27% [229/837] vs. 30%, respectively). The potential added benefit of tralokinumab was considered to outweigh this decrease in prevalence. In addition, the ≥37 ppb cut-off was similar to the value of > 35 ppb established in the classification of a subgroup of participants with particularly poor asthma outcomes [55]. Interestingly, in the STRATOS 2 trial, we observed a greater effect on AAER with tralokinumab compared with placebo in the FeNO-high subgroup than the all-comers population, but this effect was not statistically significant nor clinically meaningful [22]. Potential reasons as to why statistical significance was not achieved are discussed elsewhere [22], but may include a number of factors. For example, in addition to the low prevalence of FeNO-high participants (27%), there was a lack of opportunity to enrich the study population for a FeNO-high subgroup or to allow for stabilisation of baseline FeNO concentrations prior to randomisation. This was due to the staggered design of the STRATOS 1 and 2 trials, as FeNO was not identified as a predictive biomarker until after STRATOS 2 had enrolled participants. Further, the smaller treatment effect in terms of reduction in AAER with tralokinumab, as observed in the all-comers population in STRATOS 2, compared with STRATOS 1, may have limited the potential treatment effect in the FeNO-high subgroup. Finally, as is often done when searching for a subgroup, we selected the most appropriate subgroup from a number of potential candidates. Even though we attempted to discount for it, it is possible that we observed a random high exacerbation rate reduction within the FeNO-high subgroup in STRATOS 1.

Periostin is a matricellular protein secreted by airway epithelia in response to IL-4 and IL-13 and is involved in the development and persistence of allergic inflammation [56]. It can induce transforming growth factor-β–mediated collagen secretion from fibroblasts, which contributes to fibrosis in bronchial asthma [20, 57], and can facilitate eosinophil migration to sites of type-2 inflammation [58]. Periostin (cut-off ≥50 ng/ml) was investigated as a predictive biomarker for therapeutic effect (measured as reductions in the exacerbation rate) in Phase III trials of the anti–IL-13 mAb lebrikizumab for the treatment of uncontrolled asthma, but proved inconsistent [15]. Our analysis of STRATOS 1, using a lower cut-off value of > 27.4 ng/ml, was also able to identify a treatment effect in a periostin-high subgroup. However, this effect was less than that seen with FeNO, and was only seen for asthma exacerbations, not for the secondary endpoints based on lung function and quality of life assessments. An important limitation of periostin was the observed regional differences in baseline concentrations, as greater baseline concentrations were found in participants from the Asia/Pacific region and adolescents than other groups, which would have complicated potential use of periostin to guide personalised treatment with tralokinumab in routine practice. In contrast to the findings in relation to periostin and to those from a previous Phase IIb trial [13], DPP-4 levels were not shown to be predictive of response to tralokinumab treatment. This further highlights not only the complexity of type-2 inflammation in severe asthma, but also that the role of IL-13 in severe asthma exacerbations may be limited.

There are important strengths of our analysis. It was rigorously developed and tested using simulation exercises prior to implementation for the analysis of STRATOS 1 results. The consistency of findings across the multiple statistical methods used reassured us that the choice of FeNO with the threshold of ≥37 ppb was reasonable. One of the main limitations of analyses that aim to identify participant subgroups is the large number of individuals required [59]. For example, powering a trial to identify a 10-unit difference in treatment effect between two subgroups (of equal size) rather than powering the trial for a 10-unit treatment effect in an unselected population would require four times the number of participants [60]. The STRATOS 1 population was not large enough to fully assess the predictive properties of the assessed biomarkers because of the required sample size this would have entailed. As the trials were run largely in parallel, by the time FeNO was identified as the potentially predictive biomarker in STRATOS 1, STRATOS 2 had completed recruitment, precluding enrichment of the population of that trial with a FeNO-high subgroup. In accordance with the STRATOS 2 statistical analysis plan and based on the expected effect and sample size for the selected FeNO-high subgroup (estimated from the STRATOS 1 data), the testing strategy used in STRATOS 2 was adjusted to increase power by allowing the FeNO-high subgroup (which comprised 27% [229/837] of the participants in STRATOS 2) to be tested using the full allocated alpha. Finally, the innovative nature of SIDES could have affected the clinical team’s ability to interpret accurately the outputs in a timely manner. To help prevent this, the simulation and interpretation exercise we conducted were in part used to familiarise the clinical teams with interpretation of the data.

## Conclusion

Identifying a biomarker for predicting treatment effect of a biologic for use in severe asthma is a challenge. We describe the use of a rigorous approach using multiple statistical methods to identify a biomarker that most effectively identified a subgroup with an enhanced tralokinumab treatment effect in STRATOS 1. SIDES was applied as one of the components of this biomarker analysis plan and provided important insights into the predictive properties of the five potential biomarkers. Simulation and interpretation exercises allowed us to confirm that the methods used would be able to detect the signals required, as well as refine our ability to interpret the results and practice the decision-making process. Using data from the STRATOS 1 trial, our analyses identified FeNO at a cut-off of ≥37 ppb as the best option for predicting enhanced treatment effect to be tested in the STRATOS 2 trial. To support this finding, additional analyses were performed, including robustness and sensitivity checks to mitigate false discovery, overfitting and overoptimistic belief in the chosen subgroup. However, findings from STRATOS 2, in terms of effect of tralokinumab on AAER compared with placebo, were not sufficient to support future development of anti–IL-13 therapy with tralokinumab for severe asthma [23]. This further emphasizes the level of complication involved in subgroup identification in the severe asthma population.

## Additional files

Additional file 1: **Figure S1.** Relationship between biomarker values and number of exacerbations in the previous year in the STRATOS 1 all-comers population (full analysis set). **Figure S2.** Relationship between biomarker values and region in the STRATOS 1 all-comers population (full analysis set). **Figure S3.** Relationship between biomarker values and age categories in the STRATOS 1 all-comers population (full analysis set). (DOCX 1067 kb)

Additional file 2: Parameter modifications applied to the SIDES algorithm. (DOCX 17 kb)

## Data Availability

The data that support the findings of this study and the bespoke SIDES package that was developed to include count data are available from the authors upon written request.

## Abbreviations

AAER: Annualised asthma exacerbation rate
ACQ-6: Asthma Control Questionnaire (6-item)
AE: Adverse event
AQLQ: Standardised Asthma Quality of Life Questionnaire for 12 years and older
DPP-4: Dipeptidyl peptidase-4
FAS: Full Analysis Set
FeNO: Fractional exhaled nitric oxide
FEV_1_: Forced expiratory volume in 1 s
GAM: Generalised additive models
ICS: Inhaled corticosteroid
Ig: Immunoglobulin
IL-13: Interleukin-13
mAb: Monoclonal antibody
Q2W: Every 2 weeks
Q4W: Every 4 weeks
SAE: Serious adverse event
SC: Subcutaneously
SIDES: Subgroup Identification based on Differential Effect Search
