# Supplementary material for: Application of structured statistical analyses to identify a biomarker predictive of enhanced tralokinumab efficacy in phase III clinical trials for severe, uncontrolled asthma
Source: BMC Pulm Med. 2019 Jul 17;19:129. doi: 10.1186/s12890-019-0889-4 (PMC6637533; doi:10.1186/s12890-019-0889-4)
Supplement: Supplementary file 1 — Figure S1. Relationship between biomarker values and number of exacerbations in the previous year in the STRATOS 1 all-comers population (full analysis set). Figure S2. Relationship between biomarker values and region in the STRATOS 1 all-comers population (full analysis set). Figure S3. Relationship between biomarker values and age categories in the STRATOS 1 all-comers population (full analysis set). (DOCX 1067 kb) [file 12890_2019_889_MOESM1_ESM.docx]

Application of structured statistical analyses to identify a biomarker predictive of enhanced tralokinumab efficacy in Phase III clinical trials for severe, uncontrolled asthma

Mattis Gottlow, David J. Svensson, Ilya Lipkovich, Monika Huhn, Karin Bowen, Peter Wessman, Gene Colice

**Additional file 1**

**Fig. S1. Relationship between biomarker values and number of exacerbations in the previous year in the STRATOS 1 all-comers population (full analysis set)**


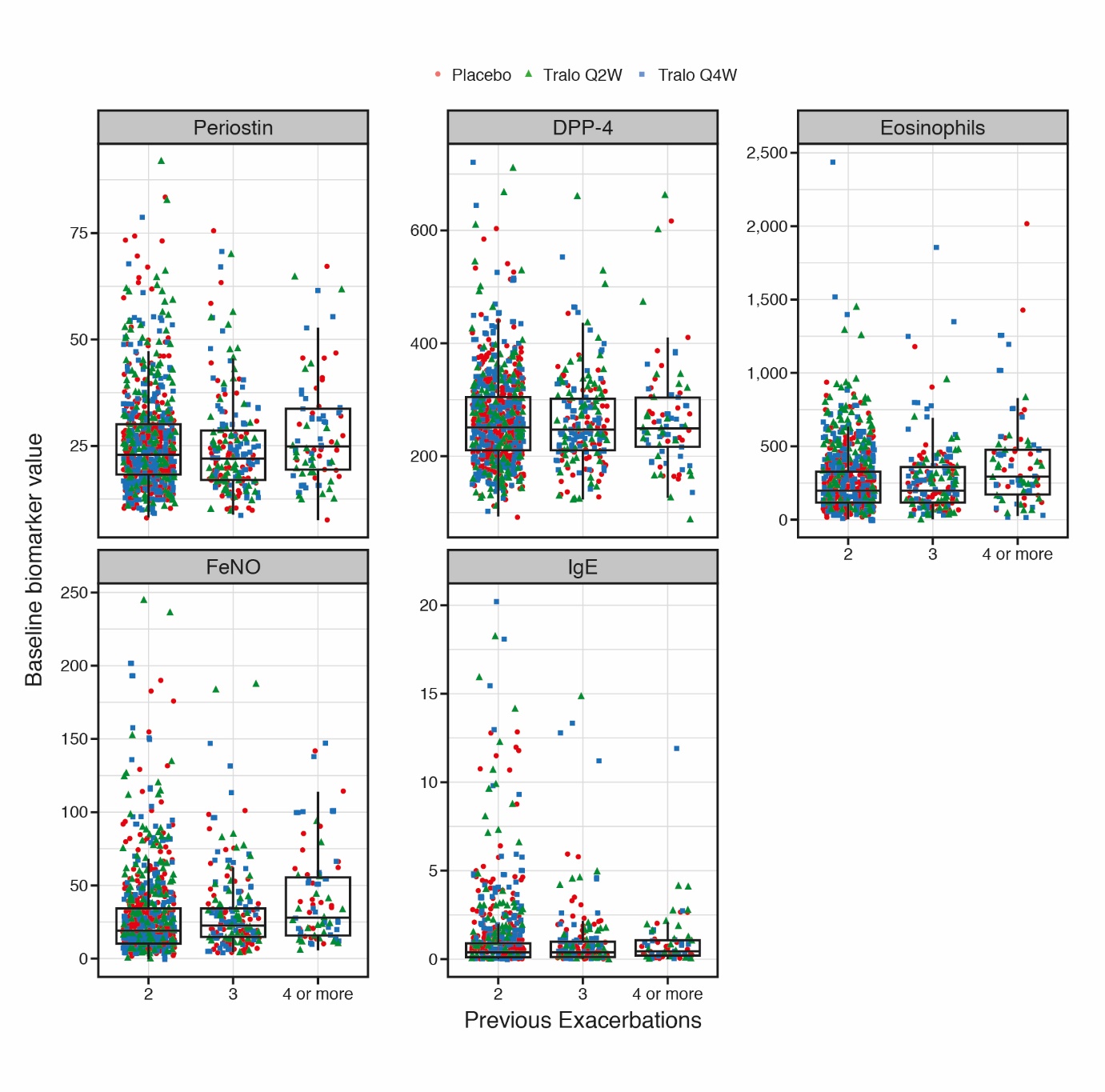


Horizontal lines in boxes show the median; the upper and lower lines show the 75th and 25th percentiles, respectively

Three subjects with outlier eosinophil values (7,510, 5,880 and 4,130 cells/μL) were removed from the plots

The placebo treatment group is a pooled treatment group (Placebo Q2W + Placebo Q4W)

Units: DPP-4 ng/mL; eosinophils cells/μL, FeNO ppb; IgE ng/mL; periostin ng/mL

DPP-4, dipeptidyl peptidase-4; FeNO, fractional exhaled nitric oxide; IgE, Immunoglobulin E; Q2W, every two weeks, Q4W, every 4 weeks; Tralo, tralokinumab

Fig. S2. Relationship between biomarker values and region in the STRATOS 1 all-comers population (full analysis set)


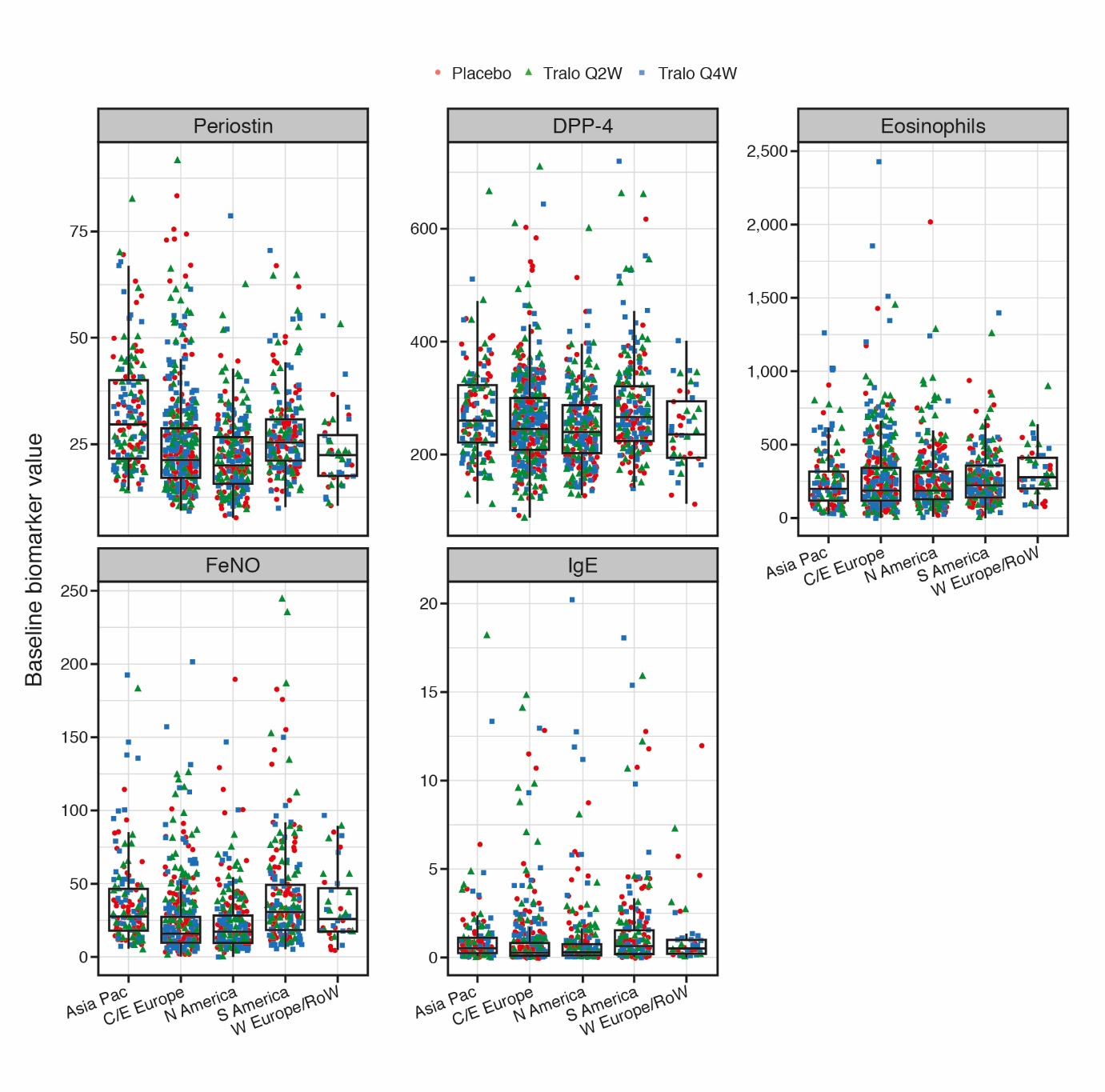


Horizontal lines in boxes show the median; the upper and lower lines show the 75th and 25th percentiles, respectively

Three subjects with outlier eosinophil values (7,510, 5,880 and 4,130 cells/μL) were removed from the plots

The placebo treatment group is a pooled treatment group (Placebo Q2W + Placebo Q4W)

Units: DPP-4 ng/mL; eosinophils cells/μL, FeNO ppb; IgE ng/mL; periostin ng/mL

C/E, Central/Eastern; DPP-4, dipeptidyl peptidase-4; FeNO, fractional exhaled nitric oxide; IgE, Immunoglobulin E; N, North; Pac, Pacific; Q2W,  every 2 weeks; Q4W, every 4 weeks; RoW, Rest of world; Tralo, Tralokinumab; W  Western

Fig. S3. Relationship between biomarker values and age categories in the STRATOS 1 all-comers population (full analysis set)


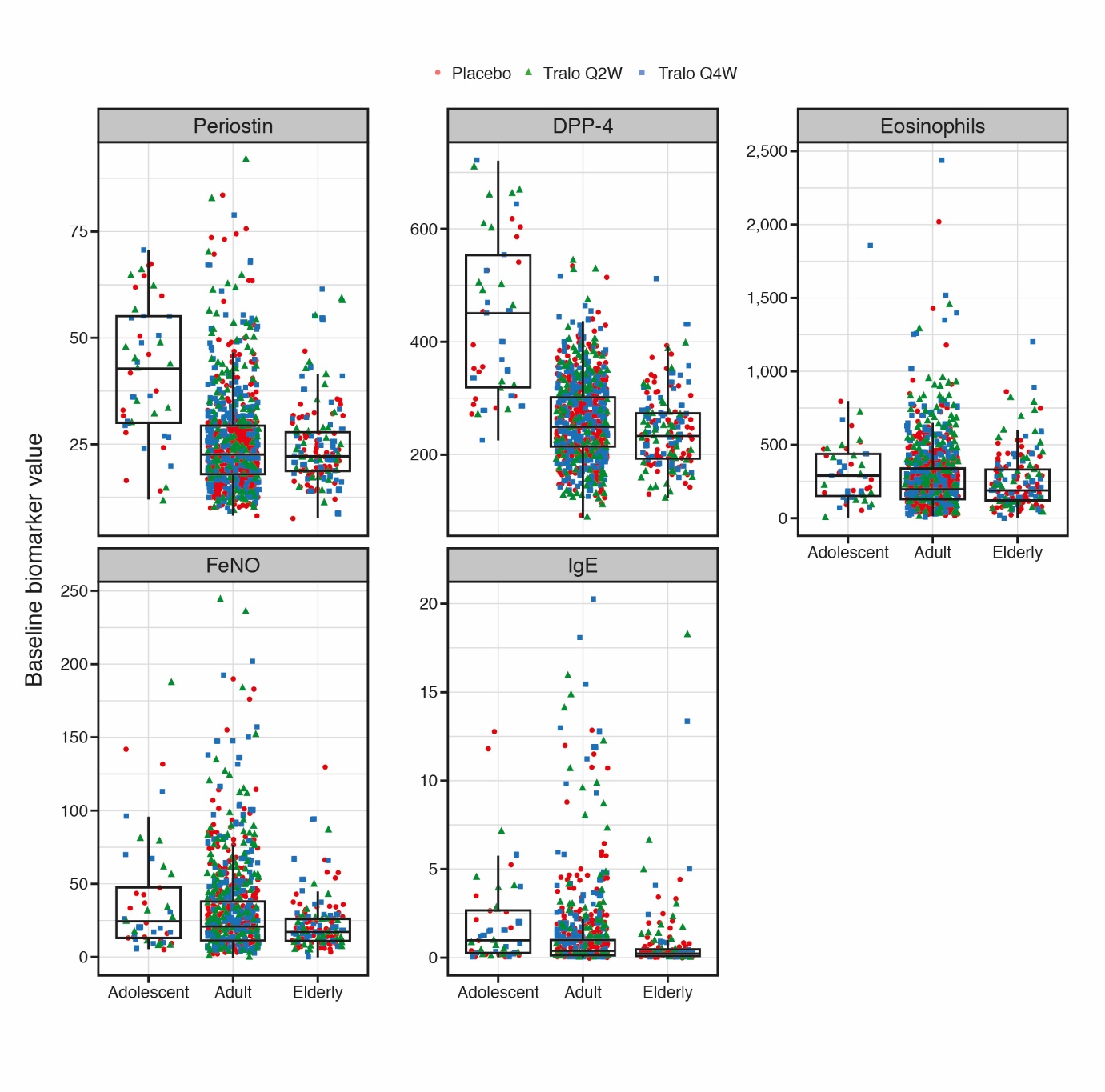


Horizontal lines in boxes show the median; the upper and lower lines show the 75th and 25th percentiles, respectively

Three subjects with outlier eosinophil values (7,510, 5,880 and 4,130 cells/μL) were removed from the plots

The placebo treatment group is a pooled treatment group (Placebo Q2W + Placebo Q4W)

Units: DPP-4 ng/mL; eosinophils cells/μL, FeNO ppb; IgE ng/mL; periostin ng/mL

DPP-4, dipeptidyl peptidase-4; FeNO, fractional exhaled nitric oxide; IgE, Immunoglobulin E; Q2W, every two weeks, Q4W, every 4 weeks; Tralo, tralokinumab
