# Supplementary material for: Application of structured statistical analyses to identify a biomarker predictive of enhanced tralokinumab efficacy in phase III clinical trials for severe, uncontrolled asthma
Source: BMC Pulm Med. 2019 Jul 17;19:129. doi: 10.1186/s12890-019-0889-4 (PMC6637533; doi:10.1186/s12890-019-0889-4)
Supplement: Supplementary file 2 — Parameter modifications applied to the SIDES algorithm. (DOCX 17 kb) [file 12890_2019_889_MOESM2_ESM.docx]

Application of structured statistical analyses to identify a biomarker predictive of enhanced tralokinumab efficacy in Phase III clinical trials for severe, uncontrolled asthma

Mattis Gottlow, David J. Svensson, Ilya Lipkovich, Monika Huhn, Karin Bowen, Peter Wessman, Gene Colice

**Additional file 2**

**Parameter** **modifications applied to the SIDES algorithm**

- Removing the restriction on subgroup size (set to at least 240 participants in the primary runs)
- Adding age, region and number of previous exacerbations to the splitting criterion model used in the search algorithm
- Adding age, previous exacerbations (2 or ≥3), body mass index, previous smoking status, previous inhaled corticosteroid use (low/medium or high), region and baseline forced expiratory volume in 1 second (FEV_1_) to the list of candidate splitters, and allowing subgroups to be based on more than one variable
- Using % change from baseline in FEV_1_ and change from baseline in Asthma Control Questionnaire-6, Asthma Quality of Life Questionnaire and symptom score as outcome variables
